# Supplementary material for: Engineering digitizer circuits for chemical and genetic screens in human cells
Source: Nat Commun. 2021 Oct 22;12:6150. doi: 10.1038/s41467-021-26359-9 (PMC8536748; doi:10.1038/s41467-021-26359-9)
Supplement: Supplementary file 1 — Supplementary Information [file 41467_2021_26359_MOESM1_ESM.pdf]

Supplementary Information for

Engineering Digitizer Circuits for Chemical and Genetic Screens in Human Cells

Nicole M. Wong[1], Elizabeth Frias[2], Frederic D. Sigoillot[2], Justin Letendre[1], Marc Hild[2,#], and Wilson W. Wong[1,#]

[1]Department of Biomedical Engineering and Biological Design Center, Boston University, Boston, MA, 02215, USA.
[2]Department of Chemical Biology and Therapeutics, Novartis Institutes for BioMedical Research, Cambridge, MA, 02139, USA.
#Corresponding authors: wilwong@bu.edu, marc.hild@novartis.com

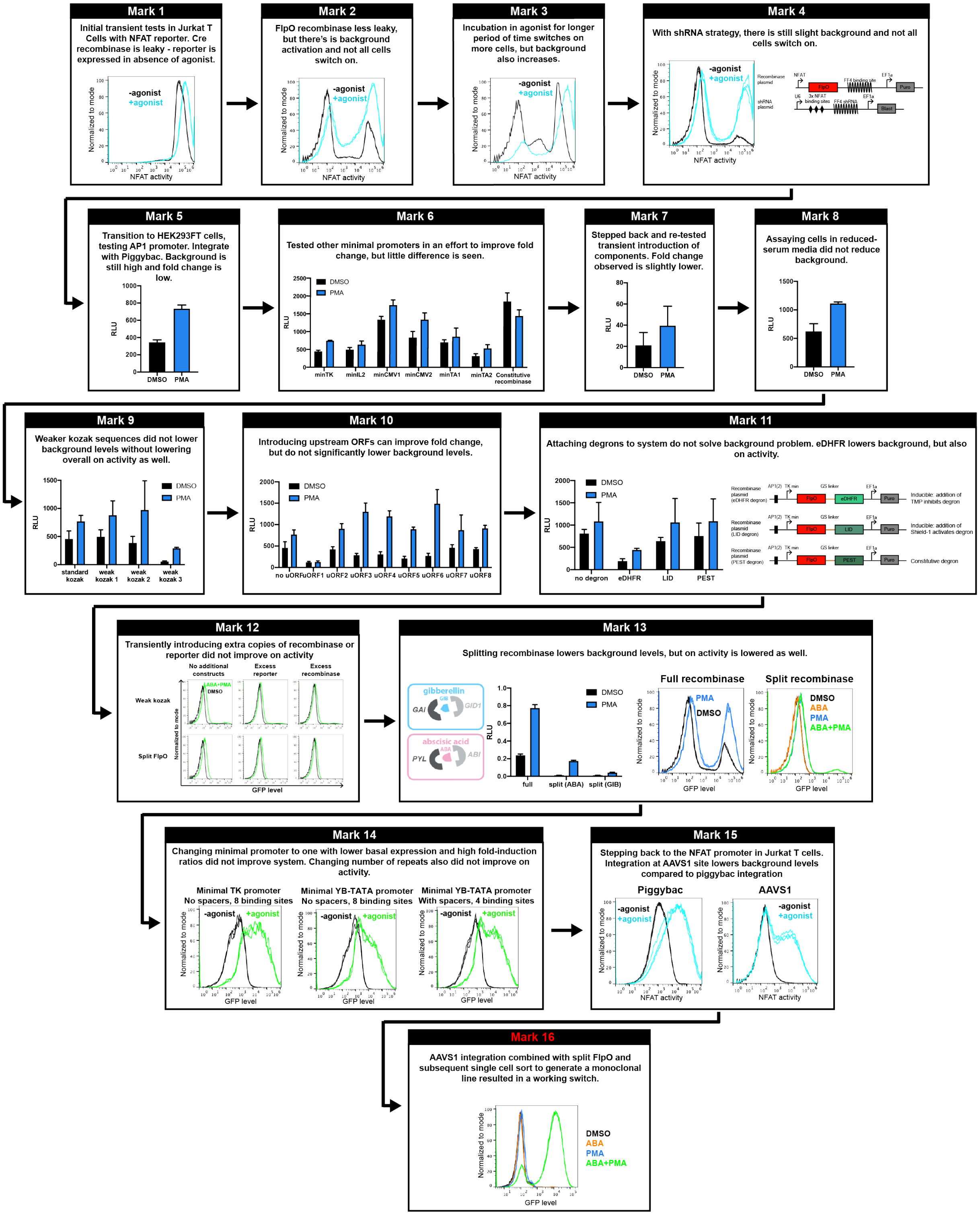

Supplementary figure 1. Evolution of RADAR. Various stages and changes made to the recombinase-based genetic circuit in order to obtain a functional reporter. Multiple approaches were explored to reduce background activity and boost "on" signal.

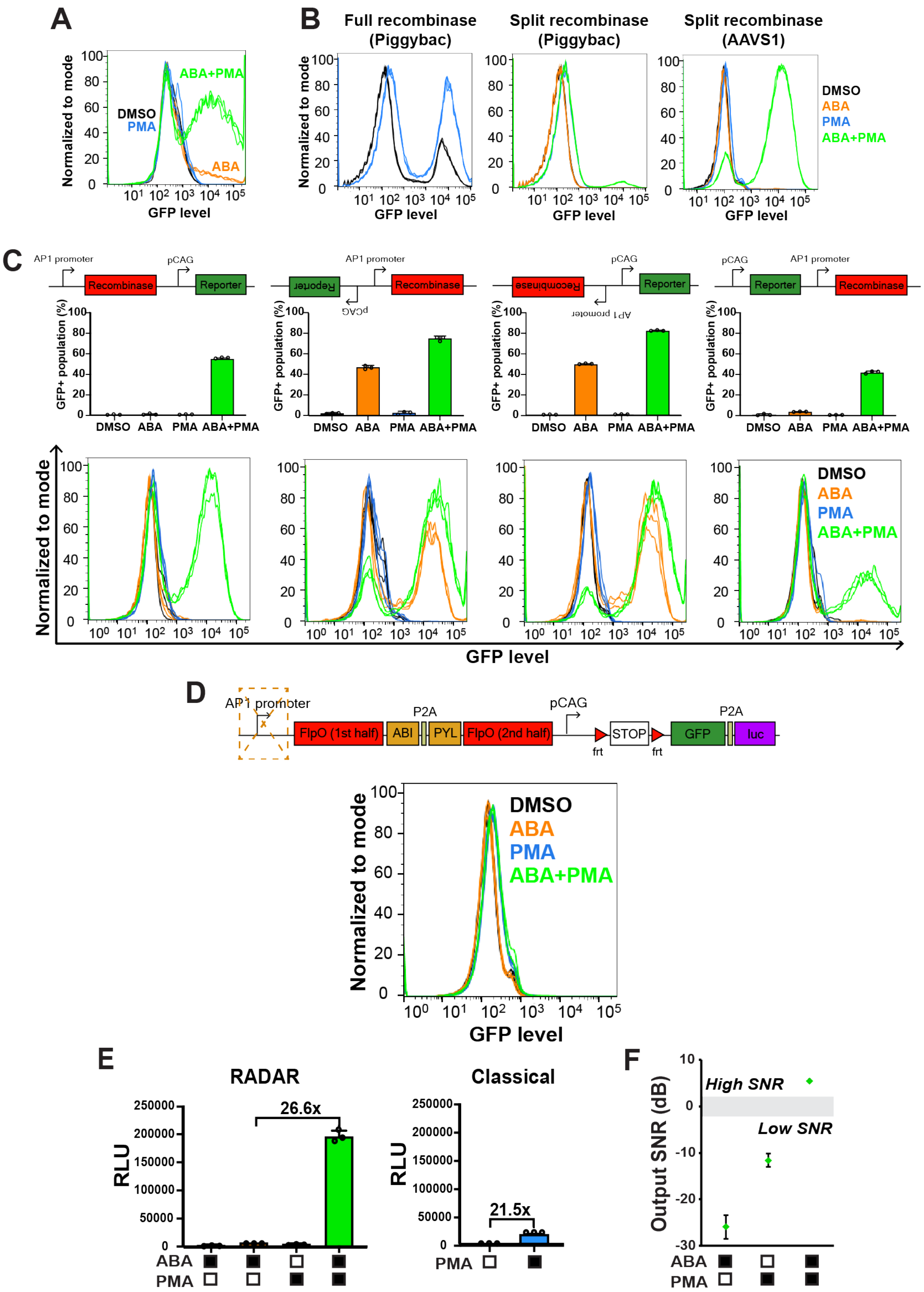

Supplementary figure 2. Further development of RADAR. A) HEK293 cells transient transfected with the recombinase-based reporter and activity measured in GFP levels. B) Major changes made to the recombinase-based reporter to generate a functional reporter. C) Various orientations of recombinase and reporter gene cassettes tested (n = 3, mean ± s.d.). D) Control

reporter missing the AP1 promoter was tested and GFP activity measured by flow cytometry. E) Comparison of luciferase levels obtained by RADAR and classical reporters (n = 3, mean ± s.d.). F) Signal-to-noise ratios for RADAR, where various conditions are compared to DMSO treatment (n = 3, mean ± s.d.).

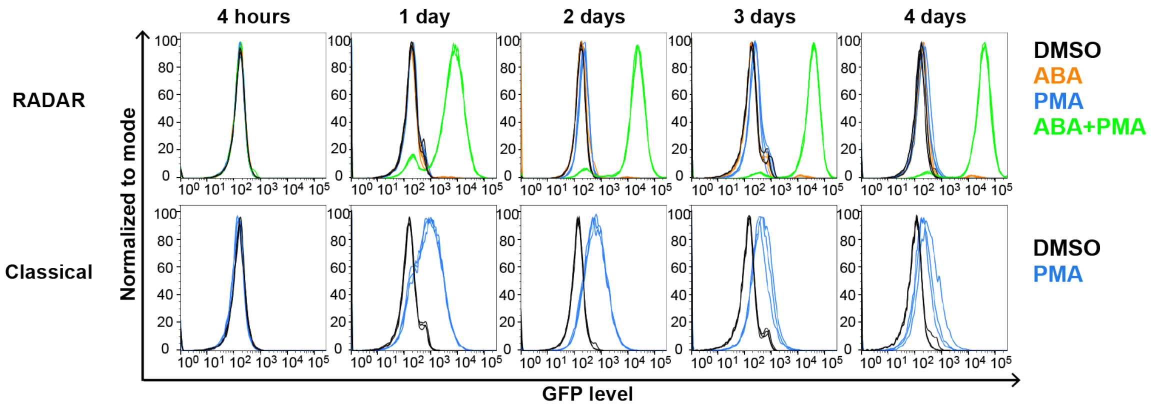

Supplementary figure 3. Retainment of memory with RADAR. Single cell data showing the retainment of GFP levels over time with RADAR as compared to the classical reporter.

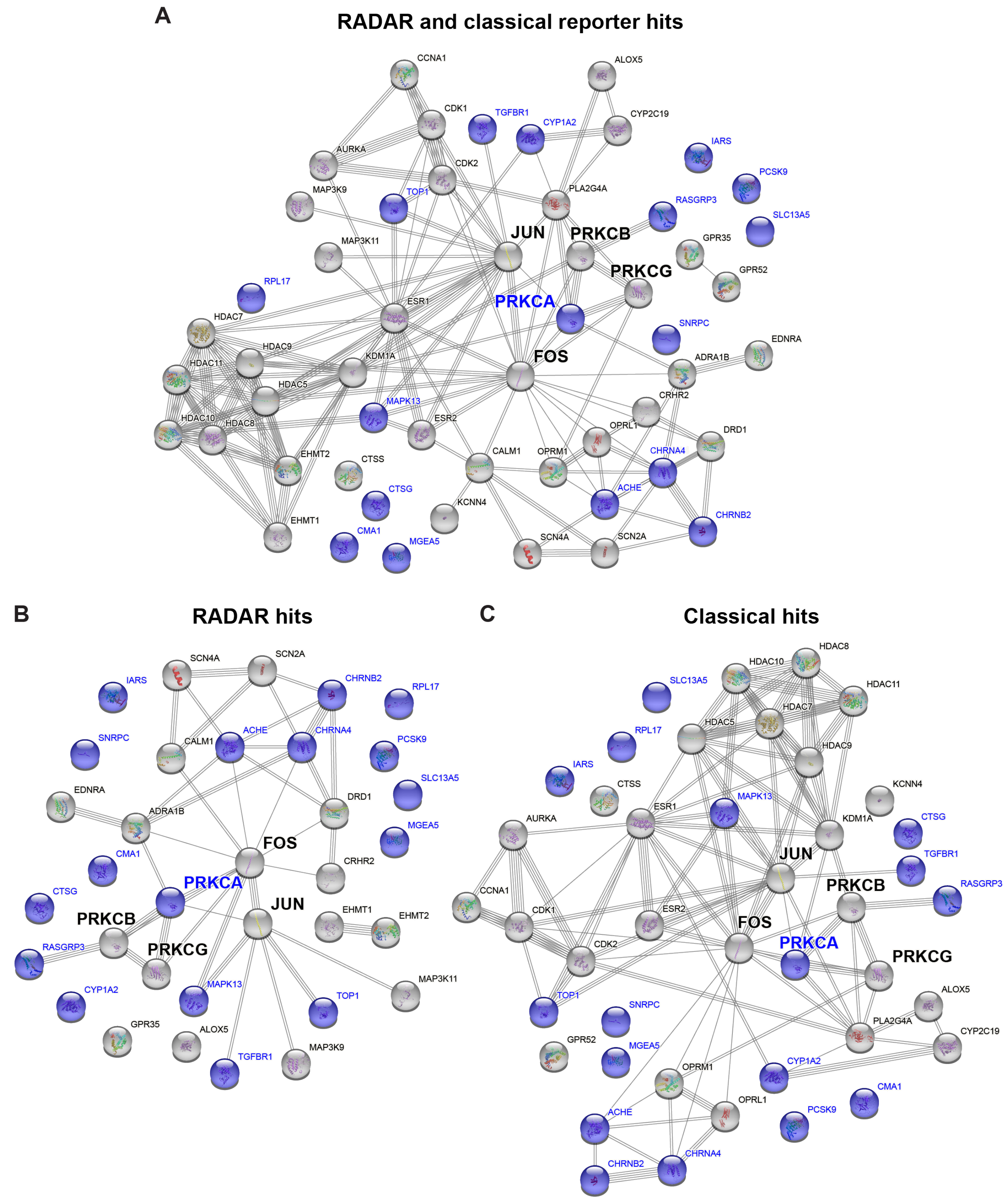

Supplementary figure 4. STRINGdb analysis of agonists from MoA library compound screen. A) Known gene targets of agonist hits from either RADAR or classical reporters were analyzed for connections to key genes in our AP-1 pathway: FOS, JUN, and conventional PKC isozymes (PRKCA, PRKCB, PRKCG). These key AP-1 genes are indicated in bold and are included for mapping reference, with the exception of PRKCA that was found to be a gene target for both reporters. Genes targets corresponding to compounds found by both reporters

are highlighted in blue. B) Known gene targets of agonist hits found by RADAR. C) Known gene targets of agonist hits found by the classical reporter. Multiple gene targets can be associated with a single compound (e.g. all HDAC genes are associated with a single compound).

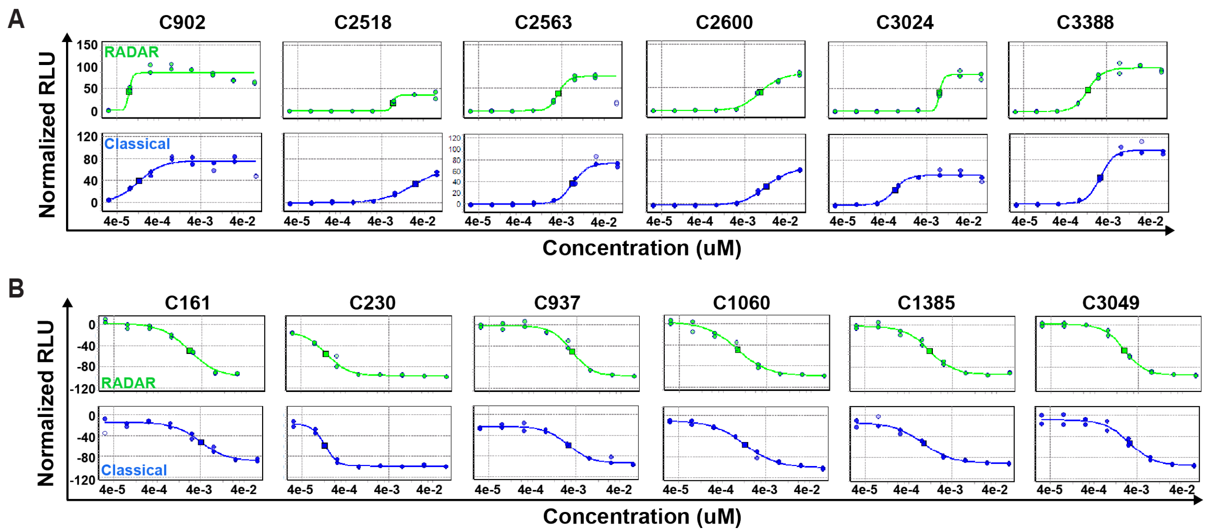

Supplementary figure 5. Comparison between dose response curves for RADAR and classical reporters in the MoA library screen. A) Dose response curves for shared agonist hits between the two reporters (RADAR in green, classical reporter in blue). Some compounds with known association to PKC (C902, C2563, C3388), and some not (C2518, C2600, C3024). Luciferase levels were normalized to positive and negative controls (n = 2). B) Dose response curves for shared antagonist hits between the two reporters. Compounds are known to target AP-1, CDK, or PKC. Luciferase levels were normalized to positive and negative controls (n = 2).

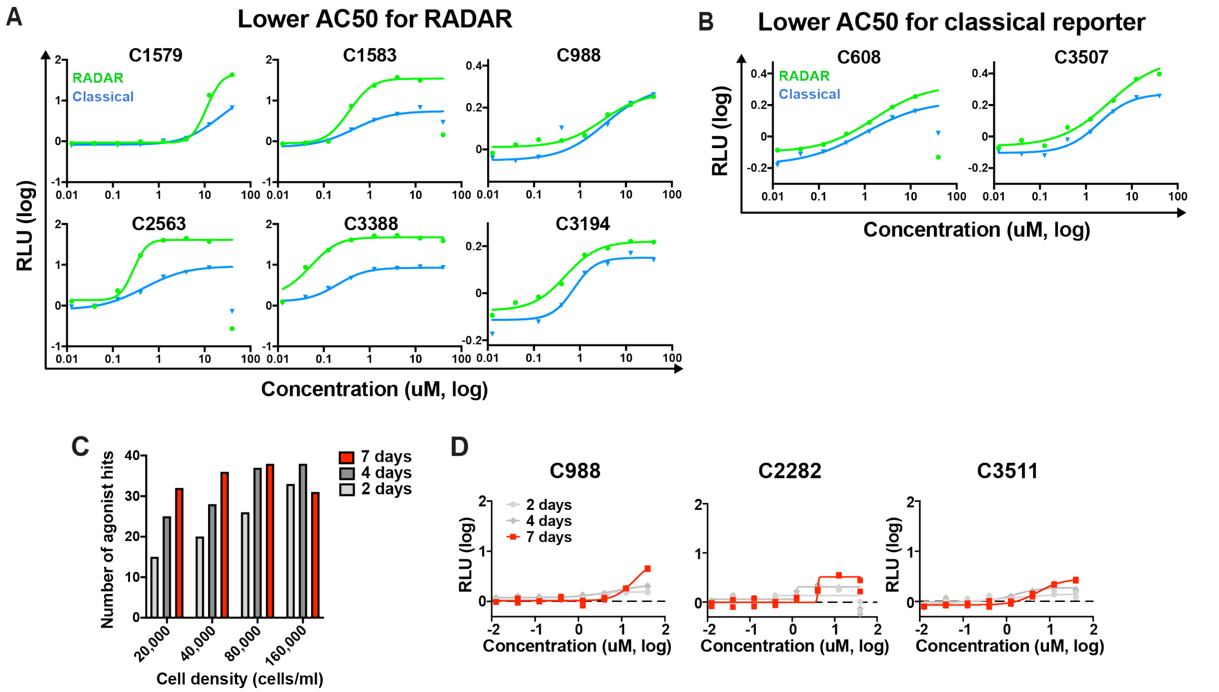

Supplementary figure 6. Characterization of RADAR sensitivity in the agonist compound reconfirmation screen. Hits were categorized by reporter AC50 values, differentiating A) dose response curves for compounds having a lower AC50 value with RADAR (green) than the classical reporter (blue) (n = 2, mean ± s.d), and B) dose response curves for compounds having a lower AC50 value with classical reporter than with RADAR (n = 2, mean ± s.d). C) Duration of compound incubation was varied in the agonist screen, and RADAR performance evaluated by the number of agonist hits detected when the reporter was incubated in the compound for 2, 4, or 7 days. Assays were run with cell densities of 20000, 40000, 80000, or 160000 cells/ml. D) Example dose response curves for agonist hits that were detected after 4 or 7 days' incubation in compound, but not 2 days'.

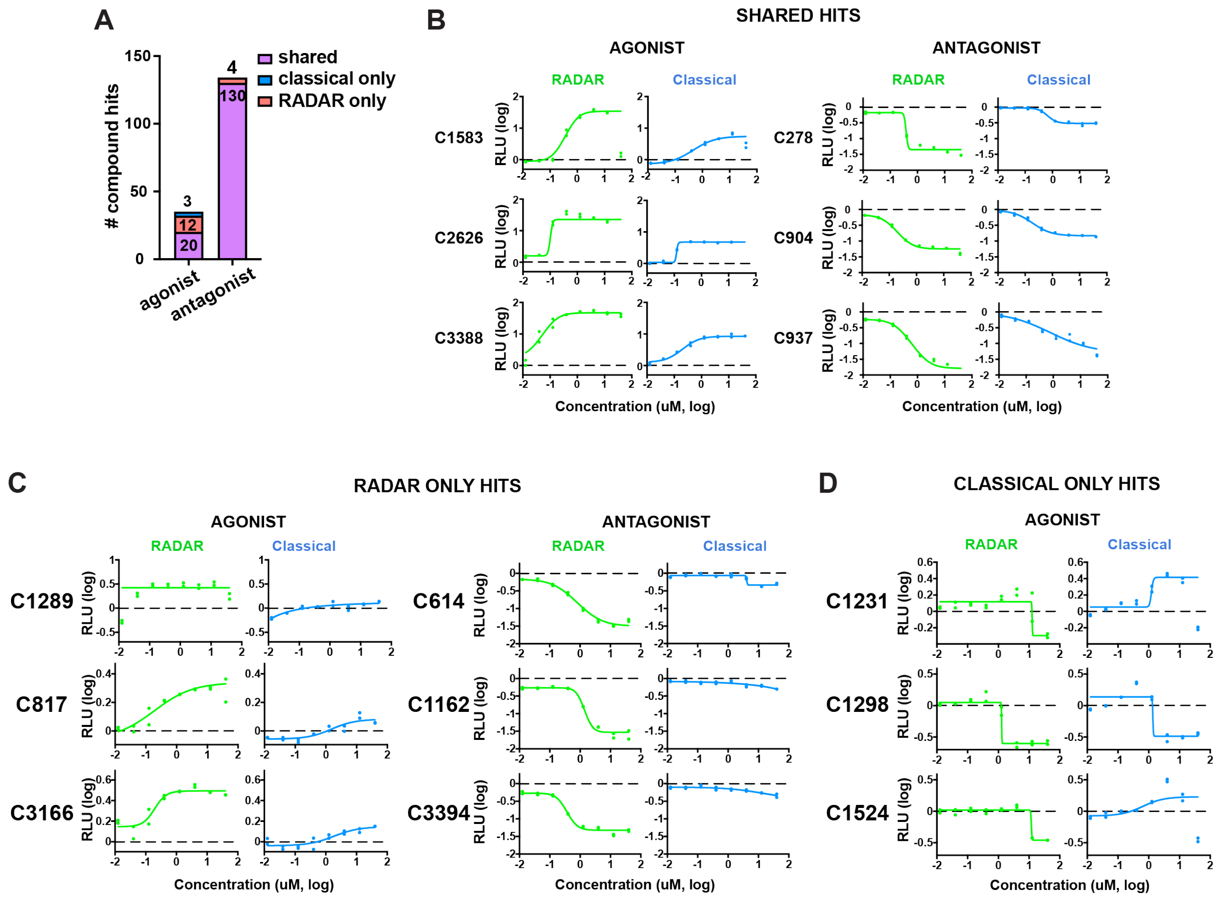

Supplementary figure 7. Compound hits from agonist and antagonist reconfirmation screens. A) Summarized number of compound hits for both RADAR and classical reporters in the agonist and antagonist reconfirmation screens. B) Example dose response curves for compound hits detected by both RADAR (green) and the classical reporter (blue) (n = 2). C) Example dose response curves for compound hits detected solely by RADAR, or D) solely by the classical reporter (n = 2).

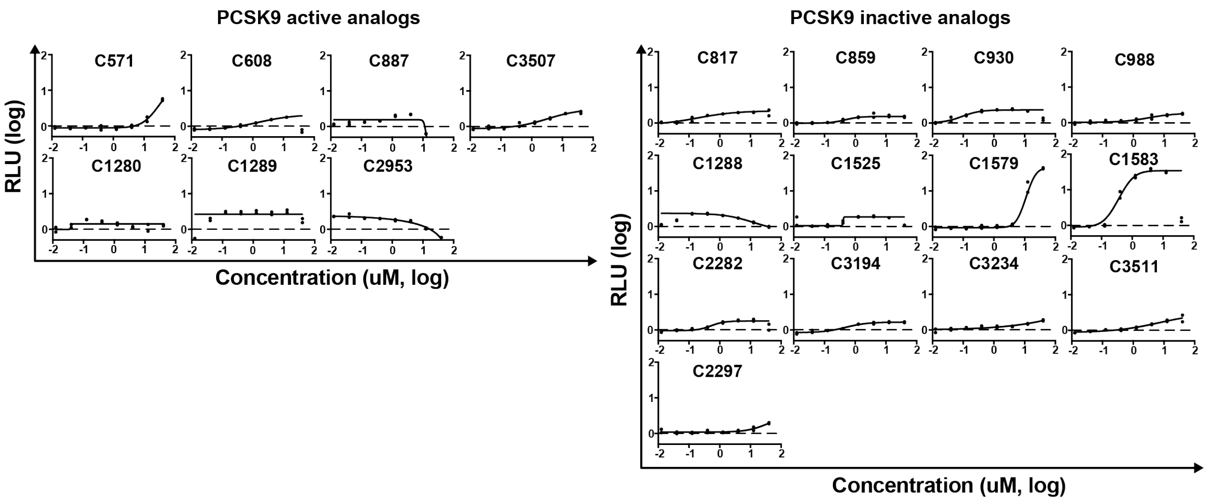

Supplementary figure 8. PCSK9-specific hits from agonist reconfirmation screen. Active and inactive analog hits for PCSK9-specific compound hit from the agonist MOA screen. Displayed are the analogs that came up as hits in the agonist reconfirmation screen (n = 2).

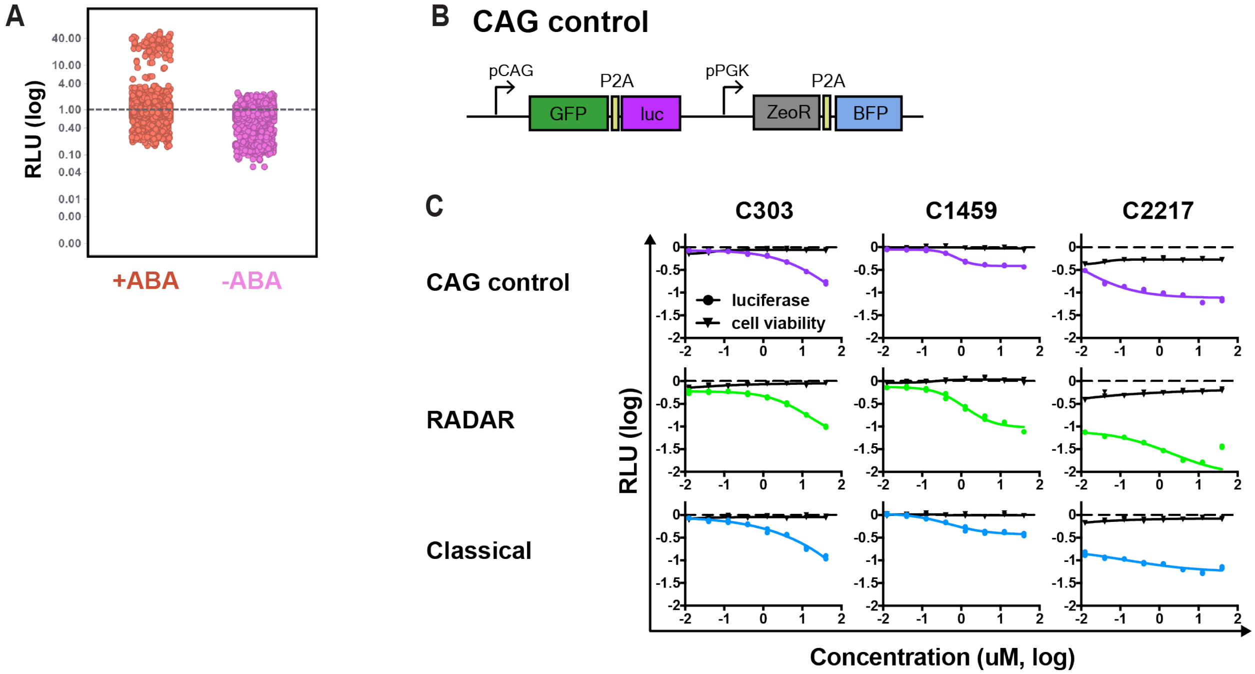

Supplementary figure 9. Controls in compound screening. A) Agonist reconfirmation screen was performed with and without ABA, and relative luciferase levels plotted. Each dot indicates treatment of cells with an individual compound in the library. B) Schematic of the constitutive CAG control reporter run with the reconfirmation compound library. C) Antagonist screen hits that emerged with CAG control, as compared with RADAR and classical reporters (n = 2).

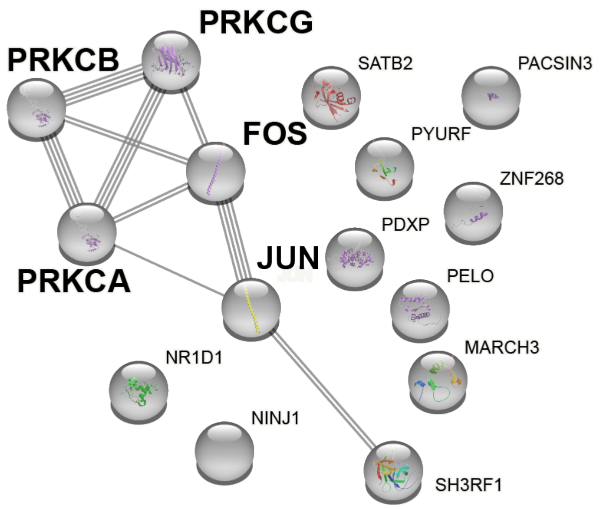

Supplementary figure 10. STRINGdb analysis of negative regulator hits from the ABA condition of the pooled CRISPR screen. Gene hits from RADAR-expressing cells treated with ABA were analyzed for connections to key genes in our AP-1 pathway: FOS, JUN, and conventional PKC isozymes (PRKCA, PRKCB, PRKCG). These key AP-1 genes are indicated in bold and are included for mapping reference.

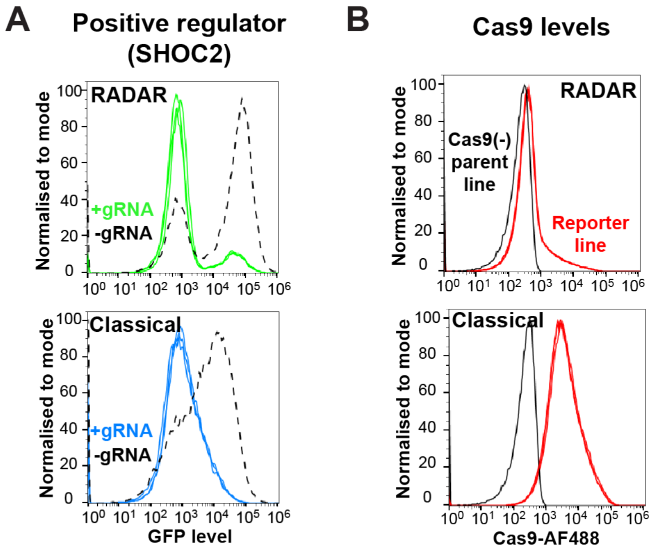

Supplementary figure 11. Single cell flow cytometry data regarding CRISPR screening and reporters involved. A) Example of a positive regulator (SHOC2) that was detected by both RADAR (green) and classical (blue) reporters. B) Cas9 levels in reporter cell lines, measured by intracellular staining (Alexa Fluor 488).

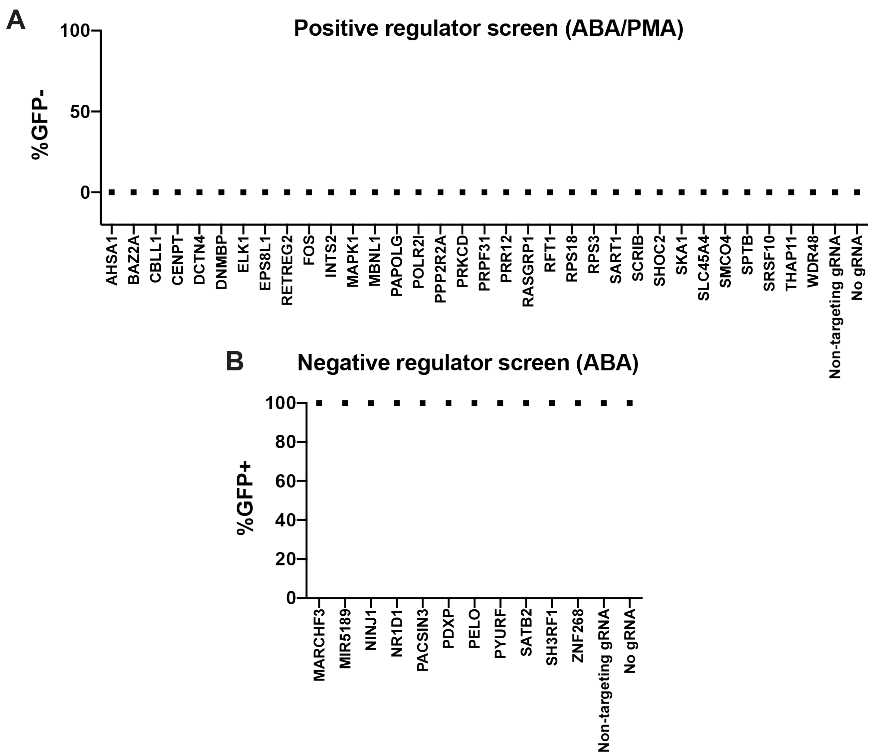

Supplementary figure 12. Behavior of constitutive CAG control in CRISPR screen reconfirmation. GFP levels when cells expressing the CAG control have genes knocked out in A) the positive regulator screen (cells treated with ABA/PMA) or B) the negative regulator screen (cells treated with ABA). GFP levels were measured in terms of percentage of GFP-negative cells for positive regulator screen, and percentage of GFP-positive cells for negative regulator screen (n = 3, mean ± s.d.).

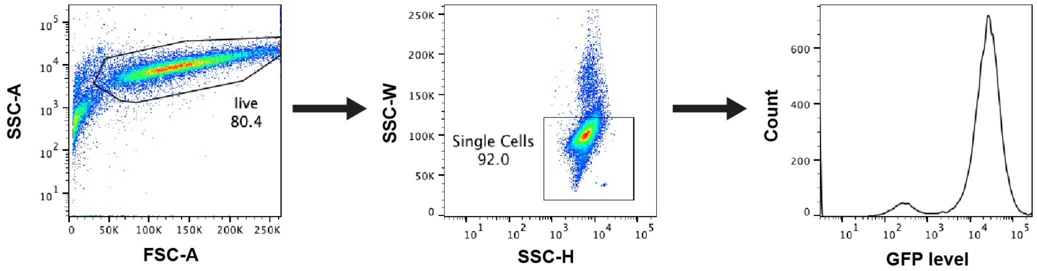

Supplementary figure 13. Example of gating for flow cytometry data. Gating strategy for flow cytometry data. Pictured is a RADAR reporter cell line post-incubation with ABA + PMA (from Figure 1C).

Primer name	Purpose	Sequence (5' to 3')
732FW	PCR Zeocin resistance gene for cloning into RADAR construct	TCCTCTTCCTCATCTCCGGGCCTTTCGATATCTCGC ACAGTGCCGCCACCATGGCCAAGCTGACAAGCGC
733RV	PCR Zeocin resistance gene for cloning into RADAR construct	TCCACGTCTCCAGCCTGCTTCAGCAGGCTGAAGTTA GTAGCTCCGCTTCCGTCCTGTTCCTCGGCCACGA
734FW	PCR BFP gene for cloning into RADAR construct	ACTTCAGCCTGCTGAAGCAGGCTGGAGACGTGGAGG AGAACCCTGGACCTATGAGCGAGCTGATTAAGGA
735RV	PCR BFP gene for cloning into RADAR construct	GATTATGATCAGTTATCTAGATTTAATTAAGCTTGTGC CCCAGTTTGCTA
825FW	PCR AP1 response element for cloning into RADAR construct	GGGCTTTTCTGTCACCAATCGCCGGCGAATTCGCGG CCGCCTTACGCGTGCTAGCTGACT
831RV	PCR AP1 response element for cloning into RADAR construct	ACGCGTCACCTTAATATGCGAAGTTTAAACTATATAC CCAGATCTTTAGTCATTAGTCAT
826FW	PCR minimal TK promoter for cloning into RADAR construct	TGACTAAAGATCTGGGTATATAGTTTAAACTTCGCATA TTAAGGTGACGCGTGTGGCCTC
832RV	PCR minimal TK promoter for cloning into RADAR construct	GAGACGAGACGAGACAGCCTGAGAATGGATGCGAGT AATGGATCTTAAGCGGGTCGCTGCAGGGTCGCT
827FW	PCR split FlpO for cloning into RADAR construct	CATTACTCGCATCCATTCTCAGGCTGTCTCGTCTCG TCTCGGCCGGCCGCCGCCACCATGAGCCAGTTCGA CATCCTG
833RV	PCR split FlpO for cloning into RADAR construct	GCTTGGATTCTGCGTTTGTTTCCGTCTACGAACTCC CAGCGTATACCGCTACAGGGCGCGTGGGGATACCC
828FW	PCR CAG promoter for cloning into RADAR construct	GCTGGGAGTTCGTAGACGGAAACAAACGCAGAATCC AAGCGCCCGGGCACTAGTTATTAATAGTAATCAATTA
834RV	PCR CAG promoter for cloning into RADAR construct	CGACCTTGATGTTTCCAGTGCGATTGAGGACCTTCA GTGCTTCGCTCGAATTAATCAATTCTTTGCCAAAATG ATGAGAC
829FW	PCR stop element for cloning into RADAR construct	GCACTGAAGGTCCTCAATCGCACTGGAAACATCAAG GTCGATTTAAATGAAGTTCCTATTCTCTAGAAAGTATA GGAACTTCCGGATTTGATCTGATCAA
835RV	PCR stop element for cloning into RADAR construct	GACTTTGCGTGTTGTCTTACTATTGCTGGCAGGAGG TCAGTCGCGAGAAGTTCCTATACTTTCTAGAGAATAG GAACTTCTCGGATTTGATCCAGACATG
830FW	PCR GFP-luciferase for cloning into RADAR construct	CTGACCTCCTGCCAGCAATAGTAAGACAACACGCAA AGTCGCCGCCACCATGGTGAGCAAGGGCGAGGAGC TGTTCACCG
836RV	PCR GFP-luciferase for cloning into RADAR construct	AGATGGCTGGCAACTAGAAGGCACAGGGTACCCCTG CAGGGATCAGTATCTTACACGGCGATCTTTCCGCCC TTCTTGGC

Supplementary Table. Primers used to clone the RADAR construct integrated into HEK293FT cells to generate the RADAR reporter cell line.
