# Supplementary material for: Engineering digitizer circuits for chemical and genetic screens in human cells
Source: Nat Commun. 2021 Oct 22;12:6150. doi: 10.1038/s41467-021-26359-9 (PMC8536748; doi:10.1038/s41467-021-26359-9)
Supplement: Supplementary file 2 — Reporting Summary [file 41467_2021_26359_MOESM2_ESM.pdf]

nature research

Corresponding author(s): Wilson W. Wong and Marc Hild

Last updated by author(s): Sep 7, 2021

Reporting Summary

Nature Research wishes to improve the reproducibility of the work that we publish. This form provides structure for consistency and transparency in reporting. For further information on Nature Research policies, see our Editorial Policies and the Editorial Policy Checklist.

Statistics

For all statistical analyses, confirm that the following items are present in the figure legend, table legend, main text, or Methods section.

n/a	Confirmed	
☐	☒	The exact sample size (*n*) for each experimental group/condition, given as a discrete number and unit of measurement
☐	☒	A statement on whether measurements were taken from distinct samples or whether the same sample was measured repeatedly
☐	☒	The statistical test(s) used AND whether they are one- or two-sided<br>*Only common tests should be described solely by name; describe more complex techniques in the Methods section.*
☒	☐	A description of all covariates tested
☒	☐	A description of any assumptions or corrections, such as tests of normality and adjustment for multiple comparisons
☐	☒	A full description of the statistical parameters including central tendency (e.g. means) or other basic estimates (e.g. regression coefficient) AND variation (e.g. standard deviation) or associated estimates of uncertainty (e.g. confidence intervals)
☒	☐	For null hypothesis testing, the test statistic (e.g. *F*, *t*, *r*) with confidence intervals, effect sizes, degrees of freedom and *P* value noted<br>*Give P values as exact values whenever suitable.*
☒	☐	For Bayesian analysis, information on the choice of priors and Markov chain Monte Carlo settings
☒	☐	For hierarchical and complex designs, identification of the appropriate level for tests and full reporting of outcomes
☒	☐	Estimates of effect sizes (e.g. Cohen's *d*, Pearson's *r*), indicating how they were calculated

Our web collection on statistics for biologists contains articles on many of the points above.

Software and code

Policy information about availability of computer code

Data collection	*Provide a description of all commercial, open source and custom code used to collect the data in this study, specifying the version used OR state that no software was used.*
Data analysis	All flow cytometry data was analyzed on FlowJo (Version 10.6.2). Data from compound screens with the MoA box were analyzed using Helios (Novartis in-house software). Data analysis for the reconfirmation compound screens was done using Spotfire (Version 10.10.3.22) and GraphPad Prism 8 (Version 8.3.1), with AC50 value calculations done using the latter software. Gene associations were analyzed on STRINGdb (Version 11.0).

For manuscripts utilizing custom algorithms or software that are central to the research but not yet described in published literature, software must be made available to editors and reviewers. We strongly encourage code deposition in a community repository (e.g. GitHub). See the Nature Research guidelines for submitting code & software for further information.

Data

Policy information about availability of data

All manuscripts must include a data availability statement. This statement should provide the following information, where applicable:

- Accession codes, unique identifiers, or web links for publicly available datasets
- A list of figures that have associated raw data
- A description of any restrictions on data availability

The data that support the findings of this study can be found in the associated supplementary data. Protein-protein interaction analysis was performed on STRING (https://string-db.org/) (Version 11.0). Chemical structures for compounds used in high-throughput screens indicated in the paper are restricted from sharing, as they are proprietary to Novartis Institutes for BioMedical Research. All other data are available from the corresponding author upon reasonable request.

Field-specific reporting

Please select the one below that is the best fit for your research. If you are not sure, read the appropriate sections before making your selection.

☒ Life sciences ☐ Behavioural & social sciences ☐ Ecological, evolutionary & environmental sciences

For a reference copy of the document with all sections, see nature.com/documents/nr-reporting-summary-flat.pdf

Life sciences study design

All studies must disclose on these points even when the disclosure is negative.

Sample size	For reporter activity assays, sample size was determined as the number of distinct wells containing a specific cell reporter that was treated with a specified drug condition. All activity assays were done in triplicate (n=3), and this was determined to be a large enough number of repeats to be representative of activity level.
Data exclusions	No data was excluded.
Replication	Reporter activity assays were performed 1-3 times, achieving similar results. Experiments other than the reporter activity assays consisted of the compound and CRISPR genome-wide screens. Due to the large scale nature of these high-throughput screens, they were each performed a single time. However, within the compound screen, compounds was tested in replicate, with multiple doses corresponding to each drug tested, and cells in the genome-wide CRISPR screen were transduced to obtain a minimum of 1000-fold representation of each sgRNA.
Randomization	Samples were not randomized. Monoclonal cell lines generated were derived from the same homogenous mix of cells.
Blinding	Experiments used cell lines that should not be affected by the experimenter, and so no blinding was used.

Reporting for specific materials, systems and methods

We require information from authors about some types of materials, experimental systems and methods used in many studies. Here, indicate whether each material, system or method listed is relevant to your study. If you are not sure if a list item applies to your research, read the appropriate section before selecting a response.

Materials & experimental systems

n/a	Involved in the study	
☐	☒	Antibodies
☐	☒	Eukaryotic cell lines
☒	☐	Palaeontology and archaeology
☒	☐	Animals and other organisms
☒	☐	Human research participants
☒	☐	Clinical data
☒	☐	Dual use research of concern

Methods

n/a	Involved in the study	
☒	☐	ChIP-seq
☐	☒	Flow cytometry
☒	☐	MRI-based neuroimaging

Antibodies

Antibodies used	Cas9 antibody (Cell Signaling #14697, 1:200), Anti-mouse Alexa Fluor 488 (Life Technologies # A11017, 1:1000)
Validation	Control cells that were positive or negative for Cas9 were stained concurrently with samples to verify antibody staining. Manufacturer's website (https://www.cellsignal.com/products/primary-antibodies/cas9-7a9-3a3-mouse-mab/14697) with linked publications indicates antibody's specificity for Cas9.

Eukaryotic cell lines

Policy information about cell lines

Cell line source(s)	HEK293FT (Thermo Fisher #R70007)
Authentication	No authentication was made. Cells were purchased directly from source.
Mycoplasma contamination	We did not test for mycoplasma contamination.
Commonly misidentified lines (See ICLAC register)	HEK-derivative HEK293FT: An easy cell line to maintain and affords high transfection efficiencies.

Flow Cytometry

Plots

Confirm that:

- [x] The axis labels state the marker and fluorochrome used (e.g. CD4-FITC).
- [x] The axis scales are clearly visible. Include numbers along axes only for bottom left plot of group (a 'group' is an analysis of identical markers).
- [x] All plots are contour plots with outliers or pseudocolor plots.
- [x] A numerical value for number of cells or percentage (with statistics) is provided.

Methodology

Sample preparation	HEK-derived cell lines were used. No processing steps were needed.
Instrument	BD LSRFortessa Flow Cytometer
Software	All flow cytometry data was collected using BD LSRFortessa software, and analyzed using FlowJo software. Compound screen was analysed using in-house software at the Novartis Institutes for BioMedical Research. Data presentation was done using GraphPad Prism and Spotfire.
Cell population abundance	Cell reporter lines were single cell sorted to generate monoclonal lines. Purity of cell lines was verified using a BFP marker indicating that the desired construct continued to be stably expressed in the cells.
Gating strategy	A polygonal FSC-A/SSC-A gate was used to remove debris from the mammalian cell populations. This was followed by gating of single cells using SSC-H and SSC-W. Cell populations indicated as "GFP-positive" are gated relative to negative controls consisting of GFP-negative cells (e.g. wild-type HEK293T or cells treated with a DMSO control).

- [x] Tick this box to confirm that a figure exemplifying the gating strategy is provided in the Supplementary Information.
