# Supplementary material for: Engineering digitizer circuits for chemical and genetic screens in human cells
Source: Nat Commun. 2021 Oct 22;12:6150. doi: 10.1038/s41467-021-26359-9 (PMC8536748; doi:10.1038/s41467-021-26359-9)
Supplement: Supplementary file 4 — Description of Additional Supplementary Files [file 41467_2021_26359_MOESM4_ESM.pdf]

Title: Supplementary data 1:
Description: Compound hits from the MOA library agonist screen using the recombinase-based and classical reporters and their known associated genes.

Title: Supplementary data 2.
Description: Compound hits from MOA library antagonist screen using the recombinase-based and classical reporters and their known associated genes.

Title: Supplementary data 3.
Description: Compound hits from agonist reconfirmation screen using recombinase-based and classical reporters and their known associated genes. Reasons for compound inclusion in reconfirmation screen are also noted.

Title: Supplementary data 4.
Description: Compound hits from antagonist reconfirmation screen performed with recombinase-based and classical reporters and their known associated genes.

Title: Supplementary data 5.
Description: sgRNA sequences for publicly available portion of the genome-wide library, and screen hits.

Title: Supplementary data 6.
Description: sgRNA sequences used in reconfirmation CRISPR screen to knock out candidate positive and negative regulators found in genome-wide pooled CRISPR screen.

Title: Supplementary data 7.
Description: RSA down, RSA up, and maximum log2(fold change) scores in the genome-wide CRISPR screen.
